# Learn from failures and stay hopeful to GPR40, a GPCR target with robust efficacy, for therapy of metabolic disorders

**DOI:** 10.3389/fphar.2022.1043828

**Published:** 2022-10-25

**Authors:** Hong-Ping Guan, Yusheng Xiong

**Affiliations:** Rezubio Pharmaceuticals Co., Ltd., Zhuhai, China

**Keywords:** GPR40 agonist, liver toxicity, diabetes, incretins, gut-restrictive

## Abstract

GPR40 is a class A G-protein coupled receptor (GPCR) mainly expressed in pancreas, intestine, and brain. Its endogenous ligand is long-chain fatty acids, which activate GPR40 after meal ingestion to induce secretion of incretins in the gut, including GLP-1, GIP, and PYY, the latter control appetite and glucose metabolism. For its involvement in satiety regulation and metabolic homeostasis, partial and AgoPAM (Positive Allosteric Modulation agonist) GPR40 agonists had been developed for type 2 diabetes (T2D) by many pharmaceutical companies. The proof-of-concept of GPR40 for control of hyperglycemia was achieved by clinical trials of partial GPR40 agonist, TAK-875, demonstrating a robust decrease in HbA_1c_ (-1.12%) after chronic treatment in T2D. The development of TAK-875, however, was terminated due to liver toxicity in 2.7% patients with more than 3-fold increase of ALT in phase II and III clinical trials. Different mechanisms had since been proposed to explain the drug-induced liver injury, including acyl glucuronidation, inhibition of mitochondrial respiration and hepatobiliary transporters, ROS generation, etc. In addition, activation of GPR40 by AgoPAM agonists in pancreas was also linked to β-cell damage in rats. Notwithstanding the multiple safety concerns on the development of small-molecule GPR40 agonists for T2D, some partial and AgoPAM GPR40 agonists are still under clinical development. Here we review the most recent progress of GPR40 agonists development and the possible mechanisms of the side effects in different organs, and discuss the possibility of developing novel strategies that retain the robust efficacy of GPR40 agonists for metabolic disorders while avoid toxicities caused by off-target and on-target mechanisms.

## Introduction

Glucagon-like peptide 1 (GLP-1) is the most successful therapy after insulin for treating diabetes and many GLP-1 receptor agonists (GLP-1RAs) have been approved in recent years (Drucker, 2018). These drugs provide robust efficacies on controlling hyperglycemia and obesity. GLP-1RAs have evolved from human endogenous GLP-1 with very short half-life, to exendin derived from lizard, chemically engineered long-lasting GLP-1RAs, and fusion protein of immunoglobulins or albumin, pegylated protein, etc. These modifications optimize the stability of GLP-1 and allow for frequencies of injection ranging from twice daily (Exenatide Byetta^®^), once daily (Liraglutide), to once weekly (Semaglutide, Tirzepatide) and potentially once bi-weekly (Rosenstock et al., 2009). By using SNAC formulation to improve bioavailability of GLP-1, oral Semaglutide (Rybelsus^®^) is available now for T2D patients, although at the expense of a higher dose, yet compromised efficacies of glucose control and body weight loss comparing with injectable Semaglutide, Ozempic^®^ (Rosenstock et al., 2019). The rapid adoption of Rybelsus^®^ since market launch reflects the patient’s need for oral treatment, even though the patients are required to avoid food and water at half hour before and after taking the medicine (Gallwitz and Giorgino, 2021). The unmet medical need for more efficacious oral medicine for T2D is very clear.

Oral drugs that increase GLP-1 levels or activate GLP-1 receptor are attractive therapies for T2D. Since approval by the FDA in 2006, sitagliptin has been the safest and efficacious antihyperglycemic agent for T2D after the first-line treatment, metformin. By sustained inhibiting dipeptidyl peptidase 4 (DPP4), the enzyme degrading GLP-1 in the blood, this class of drug elevates blood active GLP-1 level by around 1-fold and effectively lowers HbA_1c_ of T2D by 0.6–0.8% (Herman et al., 2006; Esposito et al., 2015). Majority of endogenous GLP-1 is produced in the gut (Song et al., 2019), and the enteroendocrine L-cells in the gastrointestinal (GI) tract have a great potential to secrete incretins to regulate glucose homeostasis and food intake. These incretins, including GLP-1, glucose-dependent insulinotropic polypeptide (GIP), and peptide tyrosine tyrosine (PYY), etc., act locally in the gut to slow down the gut motility, suppress appetite *via* gut-brain axis, and induce insulin secretion in pancreas, enhance fat metabolism, etc. (reviewed in Drucker, 2006, Cummings and Overduin, 2007, Gimeno et al., 2020, Richards et al., 2021; Roberts et al., 2022).

Free fatty acids (FFAs) are one of the major nutrients after a meal ingestion. Upon absorption in the gut, FFAs provide energy source, and more importantly, medium- and long-chain FFAs bind to its receptor, GPR40 or FFAR1, and regulate insulin secretion and maintain glucose homeostasis. As one of the nutrient sensors, GPR40 is expressed in the GI tract, mainly in enteroendocrine L-cells (Edfalk et al., 2008; Paternoster and Falasca, 2018). Pancreatic α- and β-cells, as well as central nervous systems and osteoclasts, also express GPR40 (Briscoe et al., 2003; Ma et al., 2007). GPR40 is a class A G-protein coupled receptor (GPCR) with conventional 7 transmembrane domains that forms ligand-binding pockets and additional eight helices with a palmitoylated cysteine at the intracellular c-terminus (Yang et al., 2021). Based on the crystal structures of GPR40 and ligand-GPR40 complex, there are two ligand-binding sites, one is the allosteric site for AgoPAM agonist (Positive Allosteric Modulation agonist) and the other is orthosteric site for partial agonist (Srivastava et al., 2014; Lu et al., 2017). Upon binding of GPR40 agonists, partial agonist induces recruitment of Gα_q_ to the c-terminal of GPCR and phospholipase C on β-cells and increases intracellular Ca^2+^ levels, which in turn causes glucose-dependent insulin secretion (Briscoe et al., 2003; Itoh et al., 2003). In contrast, binding of AgoPAM agonists to GPR40 on L-cells recruits both Gα_q_ and Gα_s_, the latter increases cellular levels of cAMP and causes secretion of incretins, including GLP-1, GIP, and PYY, etc. (Hauge et al., 2015). GPR40 agonists have been reported to have anti-inflammatory properties that may contribute to its beneficial effects on NASH (Ookawara et al., 2020), inflammatory bowel disease (Kato et al., 2019), cardiovascular inflammation (Kim et al., 2022; Lymperopoulos et al., 2022), and neurodegenerative diseases (Engel et al., 2020; Xiao et al., 2021), etc. Due to its multifactorial functions in maintaining glucose homeostasis, regulating metabolism, and reducing inflammation, GPR40 agonists had been the most popular target pursued by pharmaceutical companies for the therapy of diabetes. The journey, however, was not successful as no GPR40 agonist has been approved so far due to systemic safety concerns. Many excellent review papers have been published on GPR40 in the past decade or so (Mancini and Poitout, 2013; Mancini and Poitout, 2015; Yamashima, 2015; Governa et al., 2021), here we review recent progress of the development of GPR40 agonists, summarize the mechanisms that cause toxicities, and focus on possible strategies to resolve the side effects of GPR40 agonists while retain its efficacies. Lessons learned from the research and development of GPR40 contributed by academic and pharmaceutical fields provide valuable knowledge to tackle the problems and might direct novel strategies to resurrect this target for treatment of T2D, obesity, and other diseases.

## Clinical development of GPR40 agonists

Since the discovery of medium- and long-chain FFAs as the endogenous ligand of GPR40 and the mechanism of FFA-induced insulin secretion in β-cells *via* GPR40 activation (Itoh et al., 2003), GPR40 agonist had been actively pursued as a new therapy for T2D. The first proof of concept came from the phase II results of TAK-875, which showed a robust reduction of HbA_1c_ and fasting blood glucose with no risk of hypoglycemia after chronic treatment in patients with T2D (Negoro et al., 2010; Burant et al., 2012). In the end of 2013, Takeda announced voluntary termination of TAK-875 for T2D “due to concerns about liver safety” (Takeda News Release, 2013). This was disappointing because TAK-875 had been the new hope for T2D and no liver toxicity effect had been observed in preclinical and early clinical studies.

**FIGURE 1:**
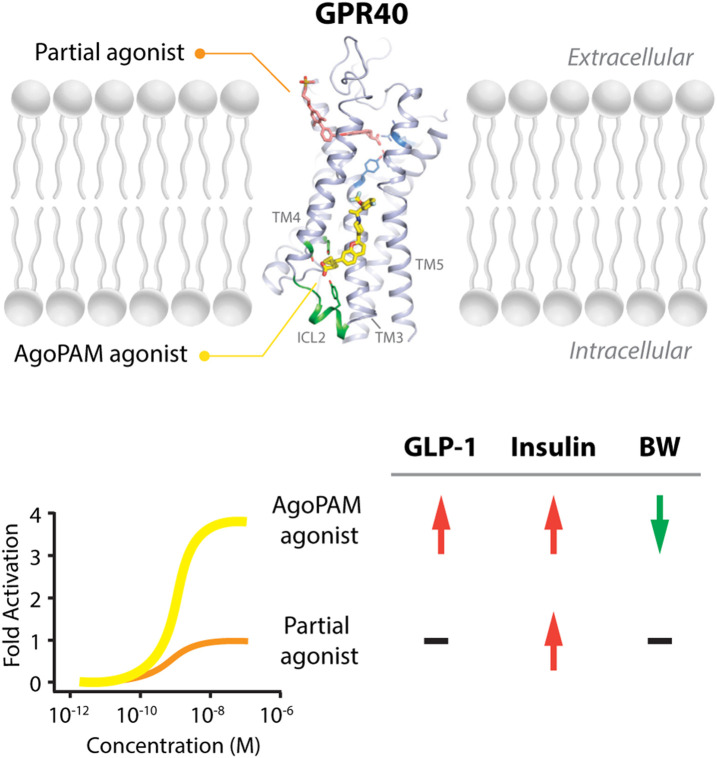
Binding sites of partial and AgoPAM agonists to GPR40 and their differential downstream effects. GPR40 partial agonists, including MK-8666 and TAK-875, bind to the area close to the extracellular surface between transmembrane (TM) helix 3 and transmembrane helix 4. In contrast, GPR40 AgoPAM agonists such as AP8 binds to an area outside of the seven transmembrane-helical bundle, in a well-defined deep pocket formed by transmembrane helices TM3, TM4, TM5 and the second intracellular loop (ICL2) (Adapted from PDB: 5TZY, Lu et al., 2017). In cells expressing low levels of GPR40, AgoPAM agonists induce robust inositol monophosphate (IP1) accumulation, around 3-4-fold higher than partial agonists. In the *in vivo* condition, GPR40 AgoPAM agonists increase GLP-1 and insulin secretion and decrease body weight, while partial agonists only increase insulin secretion.

After discontinuation of TAK-875, other companies continued developing either partial or AgoPAM GPR40 agonists. Amgen reported preclinical results of AM-1638 and AMG837, and both showed improved EC_50_ and E_max_ in cell-based assays comparing with partial agonists (Lin et al., 2011; Lin et al., 2012; Luo et al., 2012). These novel GPR40 AgoPAM agonists, also called “full” agonists, bind at a different site from that by the partial agonist like TAK-875. Functionally, these AgoPAM agonists not only induce insulin secretion in β-cells like partial agonist TAK-875, but also increase GLP-1 secretion from enteroendocrine L-cells in the gut. Altogether, it results in superior efficacies in rodent diabetic and obese models, including improved glucose tolerance and body weight loss. Merck’s AgoPAM agonist, AP8, showed robust efficacies in diet-induced obese (DIO) mice and Goto Kakizaki (GK) rats, including decreased food intake, slower GI tract motility, decreased body weight, and improved hyperglycemia. By using mouse models deficient in GPR40 and GLP-1 receptor, they proved these effects were mediated by GPR40 and induction of GLP-1 (Gorski et al., 2017; Pachanski et al., 2017). Lu et al. resolved crystal structure of human GPR40 tertiary complex with a partial agonist MK-8666 and an AgoPAM agonist AP8, and showed that partial and AgoPAM agonists bound to distinct binding pockets in the transmembrane domains and act in synergy to activate the receptor. Binding of AP8 to GPR40 involved a rearrangement of the transmembrane helices 4 and 5 and transition of the intracellular loop 2 into a short helix, which caused signal transduction of both Gα_q_ and Gα_s_ (Lu et al., 2017) (Figure 1). Meanwhile, Ueno et al. reported T-3601386, a GPR40 AgoPAM agonist, induced incretins release, suppressed food intake, and decreased body weight of rodents in a GPR40-dependent manner (Ueno et al., 2019b). Study in rats suggested that afferent vagal nerve innervation was essential for the feeding suppression induced by GPR40 AgoPAM agonist (Ueno et al., 2019a), and suppression of appetite by GPR40 agonist did not seem to require the presence of the agonist in the central nervous system.

Other companies developing GPR40 agonists include Eli Lilly, Johnson & Johnson, Bristol-Myers Squibb, AstraZeneca, Boehringer Ingelheim, etc., but all terminated their programs at different stages (Table 1), after the report of liver toxicity of TAK-875. Currently, SCO-267, CPL207280, and IDG16177 are still under clinical development based on published literatures or information. SCO-267, a GPR40 AgoPAM agonist with different backbone structure than TAK-875, was developed by Takeda and then licensed to SCOHIA. In a phase I clinical trial, SCO-267 was safe and well tolerated in healthy participants after single and multiple doses. SCO-267 stimulated insulin and incretin secretion, and robustly improved glucose tolerance in T2D patients after single dose (Nishizaki et al., 2021). No severe treatment emergent adverse events (TEAEs) were reported in the single ascending dose (SAD) and multiple ascending dose (MAD) studies. The most common TEAEs were gastrointestinal effects, including diarrhea, nausea, vomiting and decreased appetite, with a greater extent in groups receiving higher doses of SCO-267 (80 and 320 mg) compared to the placebo group (Nishizaki et al., 2021). SCO-267 is currently in phase II development.

**TABLE 1:** Selected GPR40 agonists developed or being developed by different companies.

Company	Number	Compound	Current status*	Reference
**Takeda**	1	TAK-875 (partial agonist) 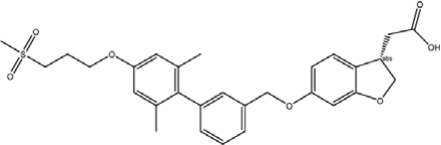	Terminated at phase III due to liver toxicity	Negoro et al., 2010
	2	T-3601386 (AgoPAM agonist) 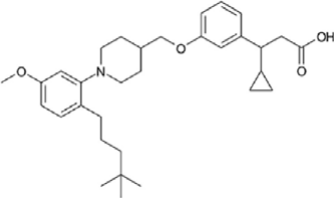	Terminated before phase I	Marcinak et al. (2018)
**Amgen**	3	AMG837 (AgoPAM agonist) 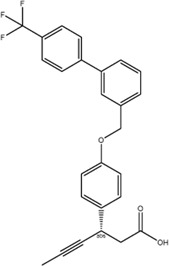	Terminated at phase I	Luo et al. (2012)
				Lin et al. (2011)
**Merck**	4	MK-8666 (partial agonist) 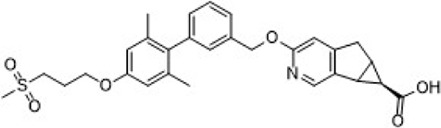	Terminated after phase I	Lu et al. (2017)
	5	AP8 (AgoPAM agonist) 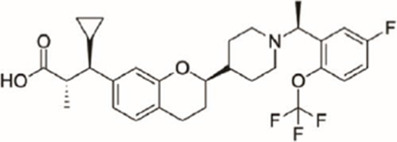		Li et al. (2017)
**Eli Lilly**	6	LY-2881835 (partial agonist) 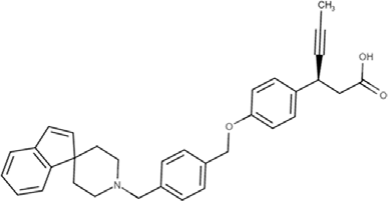	Terminated at phase I	Hamdouchi et al. (2016)
**Johnson & Johnson**	7	JNJ-4307 (AgoPAM agonist) 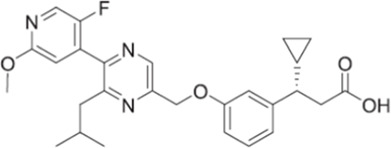	Terminated at phase I	Meegalla et al. (2018)
	8	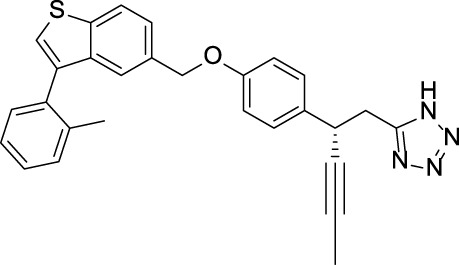	No development reported	Huang et al. (2018)
**Bristol-Myers Squibb/AstraZeneca**	9	BMS-986118 (partial agonist) 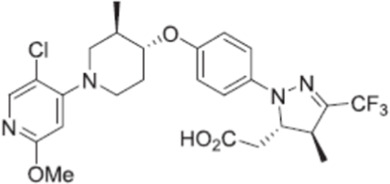	Terminated before phase I	Shi et al. (2018)
**Boehringer Ingelheim**	10	BI-2081 (partial agonist) 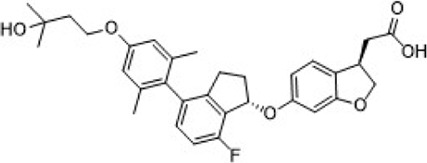	Terminated before phase I	Doerfler et al. (2020)
**Celon Pharma**	11	CPL207,280 (partial agonist) 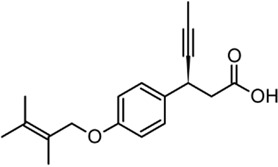	Phase II	Bazydlo-Guzenda et al. (2019)
**Ildong**	12	IDG-16177 (partial agonist) 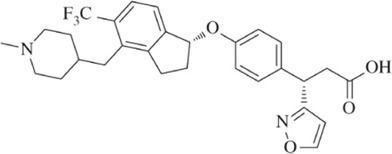	Phase I	Yoon et al. (2021)
				An et al. (2017)
**SCOHIA**	13	SCO-267 (AgoPAM agonist) 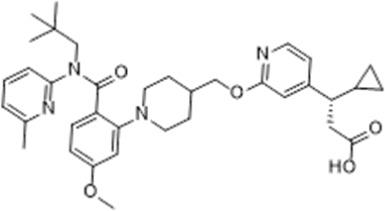	Phase II	Nishizaki et al. (2021)
**Astellas**	14	AS2034178 (partial agonist) 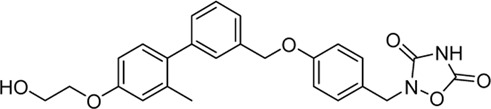	Terminated at phase I	Tanaka et al. (2013)
**Merck Sharp & Dohme**	15	MK-2305 (partial agonist) 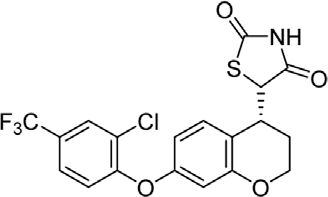	No development reported	Miller et al. (2017)
**Mochida**	16	MR-1704 (partial agonist) 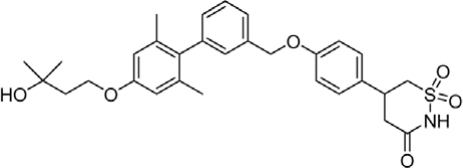	No development reported	Tsuda et al. (2017)
**Kallyope**	17	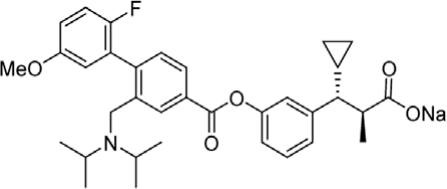	Unknown	Sebhat and He, (2020)
	18	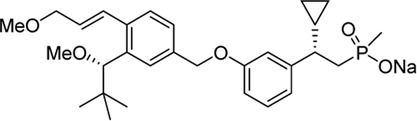	Unknown	Sebhat et al., 2021a
	19	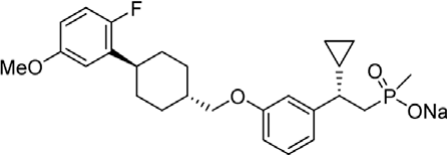	Unknown	Sebhat and He, 2021b

*Current status is based on public information. The exact developmental status of different compounds may change as programs may be resumed or terminated by developers without announcement.

CPL207280, a para-alkoxyphenylpropionic acid derivative, is a GPR40 partial agonist like TAK-875. It showed a more robust induction of insulin and improved glucose tolerance than TAK-875 in preclinical models. It also appeared to have a better safety profile than TAK-875 in rats and monkeys (Bazydlo-Guzenda et al., 2021b). Phase I trial of CPL207280 was safe and well tolerated with no serious AEs in healthy participants. No dose limiting toxicity was observed at up to 480 mg (Bazydlo-Guzenda et al., 2021c). CPL207280 is now under development in phase II trial for T2D (ClinicalTrials identifier NCT05248776, 2022). IDG16177 is another GPR40 partial agonist with different structure derived from the para-alkoxyphenylpropionic acid. Yoon et al. compared effects of IDG16177 and TAK-875 on bile acid metabolism in different *in vitro* models and claimed IDG16177 had a better safety profile and lower DILI potential than TAK-875 (Yoon et al., 2020; Yoon et al., 2021; Yoon et al., 2022). IDG16177 is now in phase I trial (ClinicalTrials identifier NCT04982705, 2021).

## Safety concerns of GPR40 agonists and possible mechanisms

### Compound-related liver toxicity

Six years after the discontinuation of TAK-875 due to liver toxicity, Marcinak et al. published the post hoc analysis results of a Liver Safety Evaluation Committee by pooling results of 15 double-blind studies of phase II and III trials, comprising 9,139 patients with T2D. They found that patients treated with TAK-875 had an increased incidence rate of serum ALT elevations in higher than 3-, 5-, and 10-fold upper limit of normal (ULN) categories compared with patients on placebo or comparator agents. Incidence of ALT levels higher than 3-fold ULN was 2.7% in TAK-875 group compared with 0.8% in comparator and 0.5% in placebo group. No dose response of TAK-875 at 25 and 50 mg daily were found for the incidence of ALT elevation. The increased ALT was resolved upon discontinuation treatment (Marcinak et al., 2018). Shavadia et al. then reported results of 7,595 patients of T2D across 8 clinical trials analyzed prior to program termination. No concerning trends were observed by local site investigators, including elevations of alanine or aspartate transaminase (ALT or AST) or hepatobiliary or gastrointestinal adverse events. However, TAK-875 treatment group showed a greater frequency of possible Hy’s law cases based on predefined liver safety measurements, and a 3- to 7-fold higher risk of ALT and AST increase. The overall probability of drug-induced liver injury (DILI) was 0.64% in TAK-875 group versus 0.06% in placebo or comparator group (Shavadia et al., 2019).

**FIGURE 2:**
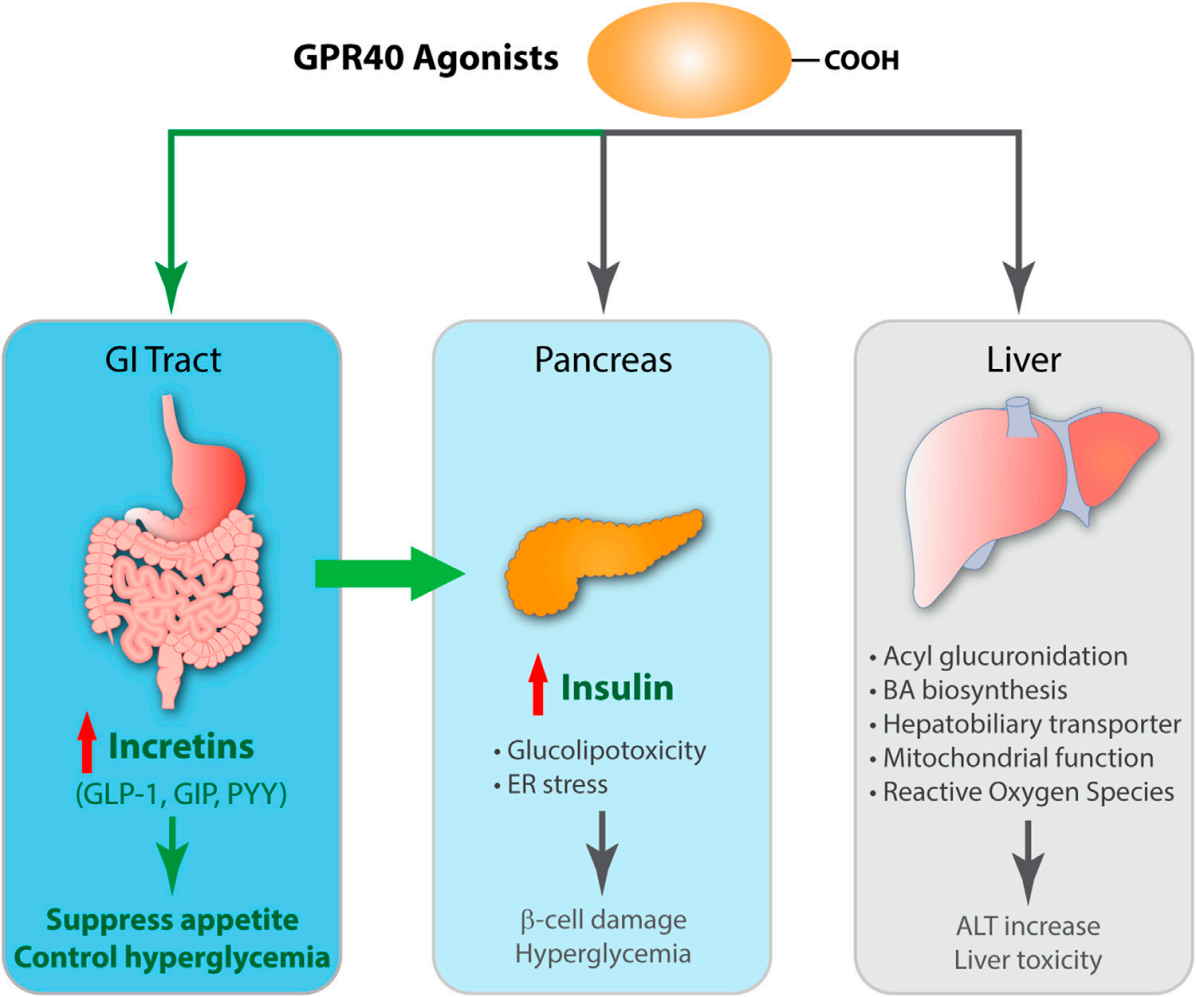
Schematic diagram of efficacy and toxicity of GPR40 agonists. GPR40 agonists, partial or AgoPAM agonists, are comprised of a large hydrophobic group with a carboxylic acid and both induces insulin secretion in β-cells by signaling transduction *via* Gα_q_ and increasing intracellular Ca^2+^ levels. AgoPAM agonists can induce both Gα_q_ and Gα_s_ pathways and cause secretion of incretins from enteroendocrine L-cells. GLP-1 induces glucose-dependent insulin secretion in β-cells, and also binds to GLP-1 receptor on vagal afferent neuron endings, which exerts suppression of appetite *via* gut-brain axis. GPR40 AgoPAM agonist causes impairment of β-cells due to ER stress and leads to hyperglycemia in rats. Many GPR40 agonists have been shown to undergo acyl glucuronidation, increase bile acid biosynthesis, affect hepatobiliary transporter, impair mitochondrial respiration, and generate reactive oxygen species. Altogether, these effects increase blood levels of liver enzymes including ALT and cause liver toxicity.

Multiple mechanisms had been linked to the liver toxicity caused by GPR40 partial and AgoPAM agonists, including acyl glucuronidation (Otieno et al., 2018; Qiang and Zhang, 2019; Shang et al., 2020; Zhu et al., 2022), inhibition of mitochondrial respiration (Otieno et al., 2018), inhibition of bile acid transporters (Li et al., 2015; Bazydlo-Guzenda et al., 2021a), generation of reactive oxygen species (Kim et al., 2018), and increase in bile acid biosynthesis (Doerfler et al., 2020) (Figure 2). As medium- and long-chain fatty acids are endogenous ligands of GPR40, carboxylic acid headgroup is a key feature of most GPR40 agonists (Table 1), which has been known in many compounds to cause idiosyncratic drug toxicities by forming reactive acyl glucuronide metabolites (Lassila et al., 2015). Otieno et al. studied the mechanism of TAK-875-induced DILI and found that TAK-875 formed covalent binding in hepatocytes *via* formation of TAK-875 acyl glucuronide (TAK-875AG), an acyl-CoA thioester intermediate. Single dose of TAK-875 at 300 and 1,000 mg/kg/day in rats increased the level of TAK-875AG in the liver, which was associated with increases in blood levels of ALT, bilirubin, and bile acids. TAK-875 also inhibited mitochondrial respiration in HepG2 cells and mitochondrial complex I and II in isolated rat mitochondria (Otieno et al., 2018). Mosedale et al. used mouse genetics to study the mechanism of TAK-875-induced liver toxicity and found that TAK-875 treatment caused changes of genes in immune responses and bile acid homeostasis, as well as oxidative stress and mitochondrial dysfunction (Mosedale et al., 2021). Doerfler et al. reported that chronic treatment of GPR40 partial agonist, BI-2081, for 4 weeks induced bile acid synthesis in rats and dogs, specifically 7a-Hydroxy-3-oxo-4-cholestenoic acid, which was correlated with increase in blood levels of liver enzymes including AST, ALT, and alkaline phosphatase (ALP) (Doerfler et al., 2020). SCO-267 was also reported to be converted to acyl glucuronide metabolites in a metabolite profiling study by using human recombinant P450 enzymes, hepatocytes, liver microsomes, as well as blood of rats dosed with SCO-267 at 10 mg/kg (mpk) (Zhu et al., 2022).

Bazydlo-Guzenda et al. performed a thorough head-to-head comparison between CPL207280 and TAK-875 in different assays to assess liver safety. CPL207280 showed improved safety profile in human hepatocytes, less inhibition on bile acid transporters, less mitochondrial injury, and no acyl glucuronidation metabolites, which is mostly contributed by differences in PK profile. PK results showed CPL207280 had higher drug levels but no accumulation in rats after repeat dose for 14 days, in contrast to accumulation of TAK-875. Chronic dosing of CPL207280 up to 250 mpk for 14 days in monkeys did not increase liver toxicity biomarkers. Similar results were obtained in ZDF rats for longer treatment at lower doses. They concluded that CPL207280 was a safer GPR40 agonist that might differentiate from TAK-875 in terms of liver toxicity (Bazydlo-Guzenda et al., 2021a). These findings are promising for CPL207280 to differentiate from TAK-875 in terms of liver toxicity, but confirmation will not be available until later stage clinical trials are finished as liver toxicities were only found in small portion of patients in phase 3 trials of TAK-875.

### Potential on-target β-cell toxicity

Acutely, binding of GPR40 agonists to receptor on β-cells induces insulin secretion *via* Gα_q_ signaling for partial agonists and Gα_q_ and Gα_s_ for AgoPAM agonists. Chronically, activation of GPR40 on β-cells causes damages, which is called glucolipotoxicity (reviewed in Brownlie et al., 2008, Poitout et al., 2010; Yamashima, 2015). By manipulating expression levels of GPR40 on β-cells, Steneberg et al. showed that knockout of GPR40 on β-cells decreased insulin secretion in response to FFAs, while overexpression of GPR40 in β-cells of mice led to impaired β-cell function, hypoinsulinemia, and glucose intolerance (Steneberg et al., 2005). Whether GPR40 partial agonists induce β-cell toxicity, however, seems to be compound dependent. Partial agonists TAK-875 and MR-1704 did not show β-cell toxicity in multiple rat studies (Tsujihata et al., 2011; Tsuda et al., 2017). In line with these results, Vilas-Boas et al. reported that chronic treatment of GW9508, a GPR40 partial agonist, did not affect β-cell physiology and function (Vilas-Boas et al., 2020).

On the other hand, Li et al. reported that following an insulinotropic effect, single dose treatment of GPR40 AgoPAM agonist caused loss of insulin secretion in rat and human β-cells *via* induction of ER stress PERK-CHOP10 pathway, and inhibition of PERK-CHOP10 pathway recued this effect. Plasma glucose of rats was acutely suppressed within the first day after GPR40 AgoPAM agonist treatment, while increased dramatically afterwards and hyperglycemia was maintained for up to 160 h. In contrast, partial agonist did not induce β-cell toxicity and hyperglycemia in the same model. This effect was abolished in GPR40 knockout rats, suggesting an on-target toxicity caused by the AgoPAM agonist (Li et al., 2017). AgoPAM agonist mediated β-cell toxicity also seems to be compound specific. In the preclinical and clinical studies of SCO-267, a GPR40 AgoPAM agonist, no β-cell toxicity had been reported in rats and humans (Ueno et al., 2019b; Nishizaki et al., 2021). Altogether, GPR40-mediated β-cell toxicity is specific for some AgoPAM agonists but not partial agonists, which has been proven in clinical trials of TAK-875 and no β-cell related adverse effects were reported in chronically treated T2D patients (Burant et al., 2012).

## Location of GPR40 protein on enteroendocrine L-cells—Luminal or basolateral?

Gut is the organ for nutrient absorption as well as chemosensing, the latter is mediated by chemosensory cells in the gastroduodenal mucosa (Psichas et al., 2015; Kaji and Kaunitz, 2017). Enteroendocrine cells, including L-cells, K-cells, I-cells, etc., comprise around 1% of the epithelium and disperse throughout the gut, which collectively form the largest endocrine system in humans (Gribble and Reimann, 2016). Enteroendocrine L-cells in the GI tract are known to express GPR40 (Paternoster and Falasca, 2018). These cells have polarity and chemoreceptors on luminal side detect specific class of nutrients and cause secretion of peptides on the basolateral side (Gutierrez-Aguilar and Woods, 2011). By using knockout animals and functional imaging of the nodose ganglion, Li et al. proved preference of sugar and fat ingestion is controlled by gut-brain axis *via* intestinal stimulation of receptors on gut epithelium and vagal nerve transmission, in which SGLT-1 controls sugar preference while GPR40/GPR120 are the essential mediators of intestinal fat signals to the vagal neurons (Li et al., 2022). Distribution of glucose sensors on enteroendocrine L-cells and the molecular mechanism involved in the regulation of GLP-1 secretion are well understood. By using isolated rat small intestine, Kuhre et al. compared the effects of vascular versus luminal glucose stimulation on GLP-1 secretion. Their results demonstrated apical SGLT-1 mediated electrogenic uptake of glucose was the major drive for glucose-induced GLP-1 secretion, and basolateral GLUT2-mediated glucose uptake with K_ATP_-channel closure secondary to intracellular glucose metabolism facilitated a full blown GLP-1 secretion (Kuhre et al., 2015). Using similar system, Brighton et al. showed that taurodeoxycholate, a G-protein-coupled bile acid receptor (GPBAR1 or TGR5) agonist, induced more robust GLP-1 secretion after vascular compared with luminal administration, suggesting the action of bile acids on GLP-1 secretion is predominantly mediated by TGR5 located on the basolateral membrane (Brighton et al., 2015).

Understanding where GPR40 protein is exactly located on the membrane of L-cells is critical for designing the gut-restricted GPR40 agonist. Christensen et al. used isolated perfused rat small intestine and found that vascular but not luminal administration of GPR40 agonists, such as linoleic acid, TAK-875, AMG 837, and AM-1638, significantly increased GLP-1 secretion to different levels (Christensen et al., 2015). This suggests GPR40 agonists need to be absorbed prior to stimulating GLP-1 secretion from enteroendocrine L-cells. Consistent with this result, stimulation of chylomicron of GLP-1 secretion in cultured primary EECs with polarity and intact tight junction is dependent on hydrolyzation of chylomicrons by lipoprotein lipase to release LCFAs (Psichas et al., 2017). Inhibiting chylomicron formation with Pluronic L-81 attenuated celiac and cervical vagal afferent activation, gut peptide secretion, and the anorectic effect induced by lipids in rats (Sakata et al., 1996; Randich et al., 2000). Although no high-resolution imaging is yet available to visualize GPR40 protein distribution on the enteroendocrine L-cells, these literatures strongly suggest that GPR40 protein is located on the basolateral membrane of enteroendocrine L-cells.

## Gut-restrictive GPR40 agonist for metabolic disorders and beyond

Given the plasticity of GPR40 ligand binding sites, orthosteric and allosteric, and multiple downstream signaling pathways involving Gα_q_, Gα_s_, and β-arrestin, a “biased agonism” approach was proposed to maximize the beneficial effects on insulin secretion and avoid possible adverse effect such as β-cell toxicity (Poitout and Lin, 2013). Mancini et al. compared signaling pathway elicited by TAK-875 and long-chain fatty acids and found the insulinotropic activity of TAK-875 was mediated by G_q/11_ and β-arrestin 2 (Mancini et al., 2015). The main challenge in this biased agonism approach is the lack of evidence that adverse effect being mediated by different signaling pathway than the beneficial effect of GPR40 activation. Furthermore, the off-target liver toxicity caused by acyl glucuronidation of carboxylic acid will not be directly changed by biased GPR40 activation.

The gut-restricted GPR40 agonist, first proposed by Poitout et al. is one approach to reduce adverse effects by significantly reducing systemic exposure of the compound (Poitout and Lin, 2013). The glucose-lowering efficacy of GPR40 agonists is mediated by two pathways, one is the activation of GPR40 receptor on β-cells and subsequent stimulation of insulin secretion, the other is the activation of GPR40 receptor on enteroendocrine L-cells and subsequent release of gut incretins such GLP-1, GIP, PYY, etc. The increased level of incretins can act on their receptors in pancreas to stimulate insulin secretion in a glucose-dependent manner. In addition, these incretins can also act locally on receptors in gut tissue to slow gut motility and suppress appetite *via* vagal nerve signaling to the brain (Ueno et al., 2019a). Body weight lowering by GPR40 AgoPAM agonists is primarily due to the action of incretins in the gut. Therefore gut-restricted GPR40 can retain full efficacy of GPR40 activation in gut tissue while avoid systemic exposure of the compound. The trade-off of this approach is the reduced glucose-lowering efficacy of the gut-restricted agonist comparing with the absorbable GPR40 agonist. Without the direct activation on β-cells, however, the glucose-lowering efficacy of gut-restricted GPR40 agonist is still very significant. Inhibition of DPP4 results in around 1-fold increase in plasma level of active GLP-1 (Herman et al., 2006), enough to lower HbA_1c_ by 0.6–0.8% in T2D patients but not enough to cause significant body weight loss, neither in animal models nor in humans (Nauck et al., 2007; Esposito et al., 2015). The GPR40 AgoPAM agonist, in contrast, has demonstrated significant body weight reduction in mouse and rat obese models (Gorski et al., 2017; Ueno et al., 2019b), indicating the level of increase in incretins is higher than achievable *via* DPP4 inhibition. In phase 1 clinical trial in T2D patients, SCO-267 increased plasma GLP-1 by 3-5 folds, GIP by 30%, and PYY by 70% after single dose and robustly suppressed glucose excursion in oGTT test (Nishizaki et al., 2021). Therefore, it is highly likely that the gut-restricted GPR40 agonist has the potential to be more efficacious than DPP4 inhibitor to control hyperglycemia in T2D patients, in addition to body weight reduction.

GLP-1 in the blood is produced by enteroendocrine L-cells and pancreatic α-cells. Both *GLP-1* and *GLP-2* genes are encoded by proglucagon gene (*gcg*), and GLP-1 and GLP-2 proteins are produced by cleavage of prohormone convertases PC1 and PC3 (reviewed in Lim and Brubaker, 2006, Sandoval and D'Alessio, 2015; Drucker et al., 2017). Since major efficacy of GPR40 AgoPAM agonists come from induced secretion of insulin and GLP-1, understanding the contributions of GLP-1 from the gut and pancreas is critical for how much a gut-restricted GPR40 agonist will preserve its efficacy. This remains a mystery till the research of Song et al., who used knockout mouse models to study the blood GLP-1 levels after deleting *gcg* gene in different segments of the gut. They quantitated protein levels of GLP-1 in pancreas and gut in normal mice and found active GLP-1 in the gut is around 100-fold higher than in pancreas. In human pancreas, the ratio of glucagon to active GLP-1 is around 50:1. By using Villin-Cre and Cdx-2-Cre system, they effectively ablated expression of *gcg* in the whole gut including small and large intestines, and in distal small intestine and colon, respectively, while preserved expression of *gcg* in other organs. Both mice had dramatically decreased plasma levels of active GLP-1 and exhibited slight glucose intolerance, although food intake and body weight were normal comparing with wild type mice (Song et al., 2019). In contrast, plasma glucagon levels only showed a trend of decrease in the whole gut *gcg* knockout mice and no change in distal gut *gcg* knockout mice compared with wild type mice, suggesting glucagon production is mainly produced from the pancreatic α-cell. Thus GLP-1 production from the GI tract, especially distal segments of the gut, is the major source of circulating GLP-1 that controls glucose homeostasis in mice. Lund et al. designed an elegant clinical study in patients undergone pancreatectomy to investigate extrapancreatic source of glucagon. Pancreatectomized patients had significantly higher baseline total GLP-1 than healthy subjects. Moreover, during glucose tolerance test comparing oral glucose tolerance test (oGTT) and isoglycemic intravenous glucose infusion (IIGI), GLP-1 response to oral glucose challenge was larger in pancreatectomized patients with a 3-fold higher peak value at 30 min post glucose administration (Lund et al., 2016). In comparison, baseline glucagon tended to be lower in pancreatectomized patients comparing with healthy participants. IIGI suppressed glucagon in both pancreatectomized patients and healthy participants. oGTT, however, induced a dramatic increase of glucagon in pancreatectomized patients, in sharp contrast to decreased glucagon in healthy participants. Increased baseline GLP-1 and more robust GLP-1 secretion in response to oral glucose challenge in pancreatectomized patients indicated that deletion of pancreas had no negative impact on circulating GLP-1 levels in humans. As such, blood GLP-1 is mainly contributed by the gut in humans. Stimulating GLP-1 secretion by gut-restricted GPR40 agonist is a practical mechanism to induce insulin secretion and suppress food intake.

As a major mechanism to induce incretin secretion in the gut, GPR40 agonists have many potential efficacies, including suppression of appetite and inducing weight loss, controlling hyperglycemia in T2D, resolving liver steatosis and inflammation, etc. (reviewed in Gimeno et al., 2020 and Secor et al., 2021). Moreover, emerging results reveal potential treatment of GPR40 agonist and GLP-1RAs for intestinal diseases, including inflammatory bowel diseases (IBD), short bowel syndrome (SBS), intestinal toxicities and coeliac disease (Hunt et al., 2021). GPR40 partial agonist AS2034178 was reported to resolve dextran sulfate sodium (DSS)-induced colitis in mice, which is mediated by induction of GLP-2 in the gut and in turn induced proliferation of epithelium and promoted healing of DSS-induced colitis (Kato et al., 2019). Indeed, long-lasting GLP-2 analogs have been approved or are being developed for SBS (Rosete et al., 2021; Naimi et al., 2022). Villumsen et al. performed a retrospective analysis of T2D patients with IBD, treatment of GLP-1-based therapies (GLP-1RAs and DPP4 inhibitors) lowered the risk of adverse clinical events related to IBD by 50% (Villumsen et al., 2021). Treatment of Liraglutide in a T-cell driven adoptive transfer colitis mouse model upregulated expression of IL-33, mucin 5b, and CCL20 in murine Brunner’s glands and improved colitis (Bang-Berthelsen et al., 2016). GLP-1RAs have shown promising efficacies in cellular and animal neurodegenerative models including Alzheimer’s disease and Parkinson’s disease (reviewed in Monti et al., 2022). Retrospective analysis of pooled clinical trials of GLP-1RAs in T2D patients for 3.6 years, including LEADER, SUSTAIN 6 and PIONEER 6, indicated that the incidence of dementia in T2D patients treated with Liraglutide or Semaglutide was 53% less than patients on placebo (Norgaard et al., 2022). Oral Semaglutide is currently being developed in phase III trial for Alzheimer’s disease (ClinicalTrials identifier NCT04777409, 2021). GPR40 agonist was reported to improve cognition and reduce β-amyloid formation in a mouse model of Alzheimer’s disease (Chen et al., 2021; Liu et al., 2021). Although the exact mechanism how GLP-1 and GPR40 agonist improve neurodegeneration is still elusive, it appears that gut-restricted GPR40 agonist might have the potential for the treatment of neurodegenerative diseases, pending the clinical results of Semaglutide for Alzheimer’s disease.

Many targets have been reported to affect incretin secretagogue in the gut (reviewed in Gimeno et al., 2020). Briere et al. compared induction of different mechanisms on circulating levels of active GLP-1 in mice, ranging from 5 to 30 pM, and the magnitude of induction was ranked from high to low as GPR40 agonist, TGR5 agonist, DPP4 inhibitor, somatostatin receptor 5 (SSTR5) antagonist (Briere et al., 2018). When combined together, these mechanisms formed a multiplicative synergy to increase active GLP-1 to unprecedented 300—400 pM, comparable to the concentrations of subcutaneously administered exenatide at 3 nmol/kg, and reached maximal efficacy in intraperitoneal glucose tolerance test (ipGTT) in wild type mice. Combined treatment of DPP4 inhibitor, GPR40 agonist, and SSTR5 antagonist dramatically increased plasma insulin and suppressed hyperglycemia in *db/db* mice. Combination of TGR5 agonist, DPP4 inhibitor, and SSTR5 antagonist showed a robust suppression in both oGTT and ipGTT in *GIP*-deficient mice. These effects were mediated by GLP-1 as blocking GLP-1 receptor abolished the efficacies (Briere et al., 2018). In line with this finding, Jespen et al. showed glucose-stimulated GLP-1 secretion was enhanced in mice deficient in SSTR5 or treated with SSTR5 antagonist (Jepsen et al., 2021), and SSTR5 antagonist and DPP4 inhibitor synergistically increased GLP-1 and improved oral glucose tolerance in mice (Liu et al., 2018; Jepsen et al., 2021). These results provide a novel strategy to maximize the potential of GLP-1 secretion in the GI tract to combat hyperglycemia and overweight. Although a rapid tachyphylaxis of GLP-1-induced deceleration of gastric emptying had been reported in humans (Nauck et al., 2011), tachyphylaxis has not been observed for either GLP-1RAs or GPR40 agonists after chronic treatments in preclinical and clinical studies (Burant et al., 2012; Burant, 2013; Gorski et al., 2017; Mehta et al., 2017; Pachanski et al., 2017; Rosenstock et al., 2019). Gut-restricted GPR40 agonist will lose the direct stimulation of insulin secretion from β-cells. With effective activation of GPR40 in the gut, however, a gut-restricted GPR40 agonist can induce secretion of large amounts of incretins including GLP-1, GIP, and PYY, and preserve sustained glucose-lowering efficacy and body weight loss, especially when combined with other mechanisms that can further increase GLP-1 secretion and prevent GLP-1 degradation.

It is advantageous to use the gut-restricted GPR40 agonist over injectable GLP-1RAs, alone or combined with other mechanism to maximize endogenous GLP-1 secretion. First, oral administration offers better compliance and probably improved glucose-lowering efficacy and body weight control in real-world patients. Although exceptionally efficacious in clinical trials, injectable GLP-1RAs, including once daily and once weekly, had less than 50% adherence after 1 year and only 30% after 2 years in real-world investigation (Weiss et al., 2020; Uzoigwe et al., 2021). Luo et al. analyzed the real-world efficacy of GLP-1RAs on body weight in around 2,400 patients with overweight or obese and T2D, 72-week treatment only showed a modest 2.5% weight loss and 30% patients lost more than 5% body weight (Luo et al., 2022). On the other hand, oral formulation of Semaglutide, Rybelsus^®^, had a rapid market uptake since its approval due to convenience of usage (Novo Nordisk financial report, 2022), although the efficacy is much less robust in both HbA_1c_ control and body weight loss than its injectable formulation (Rosenstock et al., 2019). Second, gut-restricted GPR40 agonist provides improved safety profile and therapeutic windows comparing with absorbable molecules. It is unlikely GPR40 agonist with high systemic exposure can avoid idiosyncratic toxicities in liver and pancreas. As endogenous GPR40 agonist is LCFAs, carboxylic acid in the structure of GPR40 agonists is essential for activation of the receptor and it is contained in most GPR40 partial and AgoPAM agonists (Table 1). β-cell toxicity caused by some GPR40 AgoPAM agonists is mediated by GPR40 (Li et al., 2017), thus this is an on-target toxicity and systemic exposure of GPR40 AgoPAM agonists should be minimized to avoid potential β-cell damage. Restricting drugs in the gut will effectively ablate the toxicities in the liver and pancreas. As a general practice, moreover, minimizing systemic exposure will decrease any potential off-target toxicities and improve the success rate of drug development. Third, the level of endogenous GLP-1 induced by the gut-restricted GPR40 agonist, alone or combined with other mechanisms, is far below the drug levels of injectable or oral GLP-1RAs in the GI tract, thus will have less GI tolerability issues. The C_max_ of oral Semaglutide at 20 mg (once daily) and subcutaneous Semaglutide at 1 mg (once weekly) is over 30 nM in humans (Kapitza et al., 2015; Granhall et al., 2019). In comparison, the highest GLP-1 levels induced by combination treatment were 300–400 pM (Briere et al., 2018). Fourth, there is no concern of drug-drug interaction (DDI) in the liver when the gut-restricted GPR40 agonist is combined with other mechanisms. Many fixed dose combination drugs have been approved for diabetes, such as metformin and DPP4 inhibitors, DPP4 inhibitors and SGLT-2 inhibitors, etc. Adding a gut-restricted GPR40 agonist to the existing fixed dose combination drugs will unlikely cause additional DDI as it will not be absorbed and affect liver enzymes involved in drug metabolism.

## Emerging hopes for GPR40 agonists

Improving drug molecule’s on-target potency and DMPK property reduces the drug load, and increases its safety margin. SCO-267 had superior potency in Ca^2+^ mobilization assay to AM-1638 and TAK-875 in CHO cells overexpressing human *GPR40* gene. It showed a comparable glucose-lowering efficacy in STZ rats at 10-fold lower dose than TAK-875. At this dose, the C_max_ of SCO-267 was around 40-fold lower than TAK-875 to achieve similar efficacy, indicating a possibly higher safety margin of SCO-267 and might show differentiation in terms of liver toxicity (Ueno et al., 2019b). SCO-267 demonstrated very good glucose lowering efficacies at 40 and 80 mg doses in phase I trial (Nishizaki et al., 2021), and is currently being developed in phase II trial for T2D. Whether the improvements of SCO-267 in preclinical settings can be translated to patients will be verified in later stage clinical trials, optimizing the potency and PK profile of compounds might be a plausible strategy to increase the safety margin.

Gut-restrictive design of compounds for cell surface receptors or gut intracellular targets has been pursued for many years and there have been many successful cases (reviewed in Filipski et al., 2013 and Fyfe, 2016). Here we focus on the gut-restrictive design for cell surface targets. Different approaches are available to maximize GPR40 activation in the gut while minimize systemic exposure. Traditional gut-restrictive strategy is achieved by connecting a chemical group to GPR40 agonist (the pharmacophore), to reduce cell permeability either changing the lipophilicity or simply increasing the molecular weight. For example, gut-restricted TGR5 agonists had been reported to be effectively confined in the gut to induce GLP-1 secretion, as systemic activation of TGR5 is known to stimulate excessive gallbladder filling and cause gallstones (Duan et al., 2015; Cao et al., 2016; Chen et al., 2018). The approach Shen lab used was link two TGR5 agonists together to form a large molecule (MW = 1,401), which hinders absorption in the gut (Duan et al., 2015; Cao et al., 2016). Chen et al. added a PSA probe to the pharmacophore of TGR5 agonist (MW = 761.7), which optimized the properties of the compound (RDX8940) including solubility, stability, membrane permeability, and receptor binding affinity (Chen et al., 2018). Both gut-restricted TGR5 agonists showed robust and sustained induction of active or total GLP-1, suppressed blood glucose in diet-induced mice or *ob/ob* mice, and systemic exposure of compounds as low as ng/ml range. More important, these compounds caused significantly less gallbladder filling, which might prevent gallstones formation induced by TGR5 agonists. It is known that TGR5 protein is located in the basolateral membrane of enteroendocrine L-cells (Brighton et al., 2015). How exactly these compounds reach TGR5 and activate the receptor is still elusive. The current developmental status of these two gut-restricted TGR5 agonists is not clear. Similar as TGR5, GPR40 is located on the basolateral membrane of enteroendocrine L-cells. Similar approaches could be applied to the design of gut-restricted GPR40 agonists but uncertainty remains on how efficacious the compounds will induce incretins secretion.

Acyl glucuronides formed from metabolism of drugs containing carboxylic acid, if not cleared rapidly from the body, can covalently acylate proteins and cause various idiosyncratic drug toxicities (Regan et al., 2010). To avoid the potential idiosyncratic toxicity, efforts have been made to replace the carboxylic acid group with various bioisosteres. Astellas Pharma reported an oxadiazolidinedione as the acid replacement (Tanaka et al., 2013), AS2034178, a compound that was discontinued in phase I trial (Li et al., 2020). Tetrazole as acid replacement was reported by Huang et al. A compound from this series demonstrated equal efficacy as TAK-875 in normal SD rat oGTT experiment at 10 mpk oral dose (Huang et al., 2018). Other acid bioisosteres such as thiazolidinedione (Miller et al., 2017), thiazinam-3-one 1,1-dioxide (Tsuda et al., 2017) were also reported. So far no report of ongoing clinical trials can be found for these compounds.

Soft drug design is another strategy of gut-restricted GPR40 agonists to increase the safety margin. By optimizing the metabolic stability of the compound, biologic activity of the compound in the gut is preserved while systemic exposure of the compound is much reduced due to its weak metabolic stability. A series of GPR40 agonists with soft drug design have been reported in patent applications. Compound 17 contains an ester group instead of an ether group frequently found in many GPR40 agonists (Table 1). Compound 17 is a potent GPR40 agonist with EC_50_ of less than 10 nM. At 30 min post dose of 30 mpk in mice, plasma concentration of the parent Compound 17 is <2 nM, while the ester group cleavage metabolite concentration is 27,700 nM (Sebhat and He, 2020). The rapid hydrolysis of the ester group results in very low parent drug exposure in systemic circulation, effectively reduced any potential pancreatic toxicity. The low molecular weight and more water-soluble metabolite is unlikely to generate long-lasting glucuronides and pose significant liver toxicities.

A combination of acid replacement and soft drug design comes from a series of phosphinate analogs in Compounds 18 and 19 (Table 1). The replacement of carboxylic acid group with a methylphosphinate group isostere may reduce the liver toxicity due to acyl glucuronidation, while the metabolically labile alkylphosphinate group helps reduce the systemic exposure of the parent drug. The plasma unbound concentration of Compound 18 at 2-hour post 30 mpk dose is 2.5 nM, likely due to the fast metabolism of the methylphosphinate group. This unbound drug concentration is 2- to 5-fold lower than the EC_50_ of this compound and thus pose lower risk of pancreatic toxicity (Sebhat et al., 2021a). Compound 19 is similar to Compound 18, but has slightly better ratio of EC_50_ to its unbound concentration at 2-hour post dose (Sebhat and He, 2021b). These soft drugs have the potential of activating GPR40 in the GI tract while avoiding the systemic adverse effects found in other GPR40 agonists.

## Concluding remarks

Although not successful in clinical development due to multiple safety concerns, GPR40 agonism is the most robust GLP-1 secretagogue in the gut. The mechanism of GPR40 agonists in the regulation of insulin and incretins secretion are well understood. Besides its efficacies for controlling hyperglycemia in T2D, GPR40 agonists have also been shown to be potentially applicable for other indications, including obesity and NASH, inflammatory bowel diseases, neurodegenerative diseases, etc. GPR40 plays an important role in regulating GLP-1 and other intestine incretin secretion. As a class A GPCR, it is also very amenable to small molecule intervention. Thus an oral GPR40 agonist has the potential to tap into the multiple clinically proven clinical benefits GLP-1 injectables can offer.

The causes of GPR40 agonist-induced β-cell and liver toxicity have been studied from different aspects. The most advanced active clinical study is SCO-267, which represents an approach of reducing drug load to minimize the potential liver toxicity. GPR40 agonists without carboxylic acid group are still in preclinical stage. Gut-restrictive GPR40 agonist offers the potential of reducing both the off-target liver toxicity and potential mechanism-based β-cell toxicity. Restricting the effect of GPR40 agonist in the gut could avoid these adverse effects while preserve its induction of incretin secretion and efficacies on hyperglycemia and overweight. Cutting-edge technologies, such as crystal structure-based AI screening and machine learning, single-cell sequencing, live cell imaging, etc., are envisioned to provide insights and tools for designing gut-restricted GPR40 agonists. Whether GPR40 agonist can be effectively restricted in the gut to avoid systemic exposure while retain its robust efficacies warrants further investigation. The potential of gut-restricted GPR40 agonists is attractive and might be utilized for therapy of many diseases.
